# Supplementary material for: Immunomodulation of neutrophil granulocyte functions by bacterial polyphosphates
Source: Eur J Immunol. Author manuscript; Available in PMC 2024 May 1. (PMC10666560; doi:10.1002/eji.202250339)
Supplement: Supplemental Figure 1 [file NIHMS1942041-supplement-Supplemental_Figure_1.pdf]

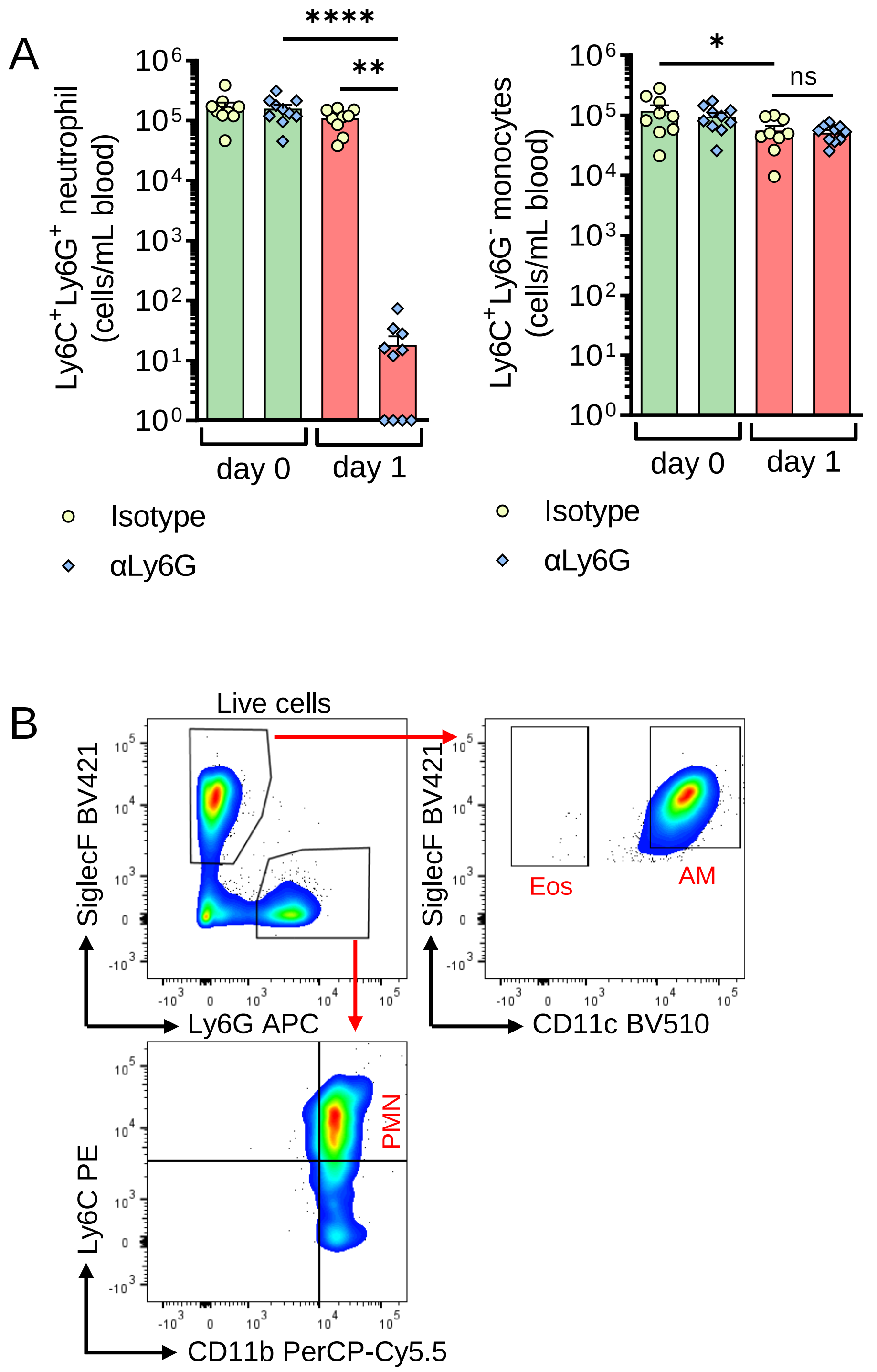

Supplemental Figure 1. Flow cytometry gating strategy and specific depletion of neutrophils. **(A)** $Ly6C^{+}Ly6G^{+}$ neutrophils and $Ly6C^{+}Ly6G^{-}$ monocytes in blood of C57BL/6J mice before (day 0) and 24h (day 1) after i.p. administration of depleting anti-Ly6G antibody (αLy6G, 100 µg/mouse) or isotype matched control antibody, flow cytometry. **(B)** Gating strategy for BALF immune cells following intratracheal (i.t.) administration of L-PolyP (40 µL at 20 mM) in C57BL/6J mice. Representative plots by flow cytometry of alveolar macrophages (AM), neutrophils (PMN) and eosinophils (Eos) identified 8h after instillation are shown. Data are representative of 2 independent experiments and are shown as mean ± SEM values from the analysis of n≥4-9 mice per group; one-way ANOVA; *$p<0.05$; **$p<0.01$; ****$p<0.0001$, ns: not significant.
